# Predicting disease recurrence in breast cancer patients using machine learning models with clinical and radiomic characteristics: a retrospective study

**DOI:** 10.1186/s43046-024-00222-6

**Published:** 2024-06-10

**Authors:** Saadia Azeroual, Fatima-ezzahraa Ben-Bouazza, Amine Naqi, Rajaa Sebihi

**Affiliations:** 1 LPHE-Modeling and Simulations, Faculty of Science, Mohammed V University in Rabat, Rabat, Morocco; 2 Faculty of Sciences and Technology, Hassan First University, Settat, Morocco; 3 LaMSN (La Maison Des Sciences Num´Eriques), Saint-Denis, France; 4 Mohammed VI University of Sciences and Health, Casablanca, Morocco

**Keywords:** Machine learning, Medical physics, Breast cancer, Recurrence prediction, Dynamic contrast-enhanced magnetic resonance imaging (DCE-MRI)

## Abstract

**Background:**

The goal is to use three different machine learning models to predict the recurrence of breast cancer across a very heterogeneous sample of patients with varying disease kinds and stages.

**Methods:**

A heterogeneous group of patients with varying cancer kinds and stages, including both triple-negative breast cancer (TNBC) and non-triple-negative breast cancer (non-TNBC), was examined. Three distinct models were created using the following five machine learning techniques: Adaptive Boosting (AdaBoost), Random Under-sampling Boosting (RUSBoost), Extreme Gradient Boosting (XGBoost), support vector machines (SVM), and Logistic Regression. The clinical model used both clinical and pathology data in conjunction with the machine learning algorithms. The machine learning algorithms were combined with dynamic contrast-enhanced magnetic resonance imaging (DCE-MRI) imaging characteristics in the radiomic model, and the merged model combined the two types of data. Each technique was evaluated using several criteria, including the receiver operating characteristic (ROC) curve, precision, recall, and F1 score.

**Results:**

The results suggest that the integration of clinical and radiomic data improves the predictive accuracy in identifying instances of breast cancer recurrence. The XGBoost algorithm is widely recognized as the most effective algorithm in terms of performance.

**Conclusion:**

The findings presented in this study offer significant contributions to the field of breast cancer research, particularly in relation to the prediction of cancer recurrence. These insights hold great potential for informing future investigations and clinical interventions that seek to enhance the accuracy and effectiveness of recurrence prediction in breast cancer patients.

## Introduction

Breast cancer exhibits the highest incidence rate among African females, accounting for 46.2% of reported cases. Furthermore, it is responsible for 39.2% of mortality in females below the age of 50 years. As of the year 2020, a total of 186,589 new cases and 85,787 deaths have been officially reported [1].

Treatment options for breast cancer include immunotherapy, radiation therapy, hormone therapy, and chemotherapy. Administering an estrogen receptor test (ER), progesterone receptor test (PR), and human epidermal growth factor receptor-2 (HER2) test can help determine which medication is the most effective. Patients with human epidermal growth factor receptor 2 (HER2), progesterone receptor (PR), and estrogen receptor (ER) deficiencies are referred to as having triple-negative breast cancer (TNBC). Ten percent to 20% of instances of breast cancer are triple negative (TNBC). Radiation therapy, chemotherapy, and surgery are often used in conjunction as part of the conventional treatment plan for TNBC [2].

In general, the terms “triple negative breast cancer (TNBC) and non-triple negative breast cancer (non-TNBC)” are used to refer to all cases of breast cancer that have all hormone receptor statuses, including hormone receptor positive, hormone receptor negative, triple negative, and triple positive. Even with recent developments in the field of breast cancer prognosis, recurrence remains a serious issue that greatly impacts mortality [3]. Predicting recurrence at diagnosis might enable optimizing treatment decisions, which may be an additional tool for medical physicists to assist in decision-making. Predictive machine learning (ML) models have been widely utilized in numerous studies [4–10]. Many of these studies have successfully demonstrated the predictive capability of utilizing clinical data in accurately predicting breast cancer recurrence.

More recent studies have incorporated radiomics as a complementary approach to augment the predictive power of machine learning algorithms, thereby further enhanc ing their ability to predict breast cancer recurrence [3, 11–14]. It is necessary to clarify the term “radiomic features,” also referred to as imaging features. In the literature, radiomic features represent a comprehensive collection of quantitative descriptors extracted from medical images that capture intricate details beyond what is perceptible to the naked eye. These features have been proven to play an intriguing role in predicting recurrent cancer, including distant metastasis, local recurrence, and locoregional recurrence, as evidenced by multiple studies [15–17].

When considering recurrent breast cancer, the integration of radiomics with machine learning techniques holds promise as a key approach for developing precise and accurate treatment plans in radiation therapy. This is primarily due to their ability to provide valuable imaging phenotypes, offering deeper insights into the tumor characteristics and facilitating personalized treatment strategies.

In this study, our objective was to forecast the likelihood of a recurrence of breast cancer across a highly diverse group of patients, representing a range of cancer types and stages, including triple-negative breast cancer (TNBC) and non-triple-negative breast cancer (non-TNBC).

To achieve this, we employed three distinct models. The first model, referred to as the clinical model, focused on utilizing machine learning algorithms with exclusively clinical and pathology data. The second model, known as the radiomic model, centered on machine learning algorithms specifically designed to analyze dynamic contrast-enhanced magnetic resonance imaging (DCE-MRI) features. Lastly, the merged model aimed to combine the strengths of the clinical and radiomic data by integrating both datasets. By employing these three models, we aimed to explore the predictive capabilities of machine learning in the context of a diverse patient population, encompassing both TNBC and non-TNBC cases. These models provided us with the opportunity to assess the individual contributions of clinical data, radiomic features, and their combined effects on predicting breast cancer recurrence accurately.

### Related works

Using machine learning models in conjunction with clinical and radiomic characteristics, a number of research have been carried out to investigate the prognostication of disease recurrence among individuals suffering with breast cancer. In order to predict the chance of a recurrence of breast cancer, Alzu’bi et al. [6] in Jordan, used machine learning techniques in their study. The goal of the researchers’ natural language processing system was to extract important information from King Abdullah University Hospital’s computerized health records. The construction of a specific medical dictionary centered on breast cancer was made easier by the integration of these elements. Expert medical professionals evaluated the retrieved data after it had been analyzed using a variety of machine learning techniques, confirming the correctness of the projected results. The study’s conclusions on the effectiveness of machine learning algorithms in predicting breast cancer recurrence were presented, with the OneR algorithm being shown to be the most effective in terms of obtaining a desirable trade-off between sensitivity and specificity. The created medical lexicon has the potential to help doctors make quick and educated decisions about therapy, which will support the use of customized medicine techniques in the management of breast cancer.

In the aforementioned investigation [18], machine learning and ensemble learning methodologies were used to predict the probability of recurrence in patients with breast cancer. Through the examination of an extensive dataset obtained from The Cancer Imaging Archive, we integrated various types of information, including demographic, clinical, pathology, genomic, and treatment data, in order to construct prediction models that exhibit an outstanding degree of accuracy. The methodology employed in our study encompassed the utilization of feature selection and Synthetic Minority Over-sampling Technique (SMOTE) to mitigate the issue of data imbalance and identify pertinent features. Among the various methods utilized, the Extreme Gradient Boosting (XGBoost) exhibited the most superior predictive capability in relation to recurrence. The implications of these findings are of great importance in enhancing treatment planning and mitigating the likelihood of treatment failure among individuals with breast cancer. Rana et al. [19]. utilized machine learning techniques in a separate investigation centered on the diagnosis of breast cancer and the prediction of its recurrence. In the current study, the effectiveness of four machine learning algorithms was compared. The findings indicated that support vector machines (SVM) exhibited notable efficacy in predictive analysis, whereas K-nearest neighbors (KNN) demonstrated superior performance in accurately predicting the recurrence and non-recurrence of malignant cases. The research underscored the potential of machine learning in the automated diagnosis of breast cancer and emphasized the significance of precise and timely detection.

The researchers conducted a study in which they investigated the prediction of breast cancer using ensemble machine learning algorithms [20]. The study conducted an analysis on a dataset pertaining to breast cancer, with a specific focus on investigating risk factors including family history, physical inactivity, psychological stress, and breast size. The prediction task utilized two widely recognized ensemble algorithms, namely random forest and Extreme Gradient Boosting (XGBoost). The analysis encompassed a total of 275 instances, each characterized by 12 distinct features. The findings demonstrated that the random forest algorithm achieved an accuracy rate of 74.7%, while XGBoost achieved an accuracy rate of 73.63%. These results highlight the potential of ensemble techniques, such as random forest and XGBoost, in the prediction of breast cancer.

In another study, the researchers aimed to investigate the role of MRI-based radiomics features in predicting the risk of tumor recurrence in patients with ER + /HER2 − invasive breast cancer who underwent Oncotype DX testing [21]. A total of 62 patients were included in the analysis. Radiomics features were extracted from both the tumor and the surrounding peritumoral tissues. The multivariate machine learning algorithm used was partial least square (PLS) regression. The top 5% of radiomics features with the largest PLS *β*-weights were selected for the analysis. The performance of the radiomics model was evaluated using leave-one-out nested cross-validation (nCV) and receiver operating characteristic (ROC) analysis. The results showed that the radiomics model achieved an area under the curve (AUC) of 0.76, indicating its potential to accurately predict the risk of tumor recurrence in early ER + /HER2 − breast cancer patients. Additional examination that integrates particular dynamic contrast-enhanced (DCE) images also revealed a propensity towards statistical significance. The results indicate that utilizing a radiomics-based machine learning methodology shows potential in forecasting the likelihood of recurrence in this specific group of patients.

In a recent study with a similar focus [22], researchers developed machine learning models to predict disease recurrences in breast cancer patients who underwent surgery. They utilized clinical data and radiomic characteristics obtained from 2-deoxy-2-[18F]-fluoro-d-glucose positron emission tomography ([18F]-FDG-PET) scans. The study included 112 patients with 118 breast cancer lesions, divided into training and testing cohorts. By employing various machine learning algorithms, including decision tree, random forest, neural network, k-nearest neighbors, Naive Bayes, Logistic Regression, and support vector machine, the researchers achieved promising results. The models, which integrated clinical and radiomic data, exhibited a notable level of precision and attained area under the receiver operating characteristic curves (AUCs) surpassing 0.80. The present study emphasizes the potential of machine learning methodologies in the prediction of disease recurrences and the provision of support in the postsurgical management of breast cancer patients.

## Methods

### Dataset

The Duke-Breast-Cancer-MRI [23] from the publicly accessible TCIA (The Cancer Imaging Archive) [24] database provided the data used in this investigation.

**Fig. 1:**
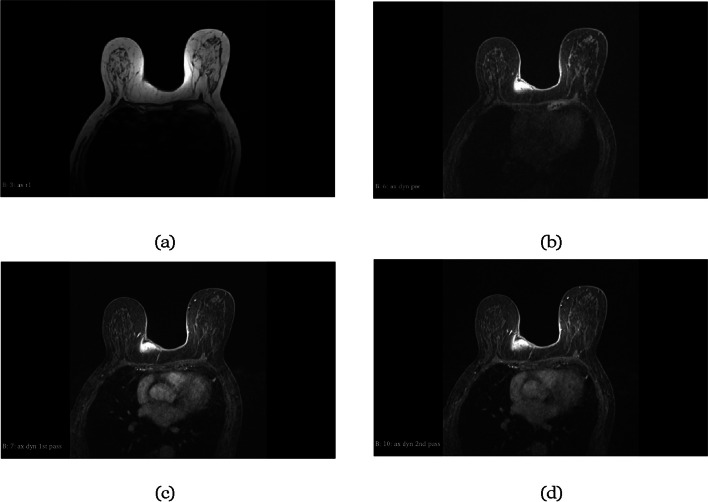
Preoperative DCE-MRI breast tumors from Saha et al. [23]. Example cases of DCE tumor images. **a** Nonfat-saturated T1-weighted sequence. **b** Fat-saturated gradient echo T1-weighted pre-contrast sequence. **c** 1st post-contrast sequence. **d** 2nd post-contrast sequence

The dataset comprised 922 patients diagnosed with invasive breast cancer, whose biopsies were confirmed, and were collected from a single institution between January 2000 and March 23, 2014. All participants underwent axial breast magnetic resonance imaging (MRI) (Fig. 1). A majority of patients, specifically 58%, were diagnosed with non-triple-negative breast cancer (TNBC), while a smaller proportion, specifically 17.79%, were diagnosed with TNBC. The cohort used in this study is heterogeneous [25]. Patients were scanned in different ways, which means that there is variability in the imaging acquisition parameters, which makes the study more general. Technical information of MRI was excluded expect days from diagnosis to MRI, field of view, and repetition time information. The data could be classified into two sets: (1) Demographic, clinical, pathology, and treatment data and (2) imaging features: 592 imaging features were extracted from the tumor and the surrounding tissue. The features extracted pertain to “(a) the volume of breast and FGT, (b) size and morphology of the tumor, (c) enhancement of the FGT, (d) enhancement of the tumor, (e) combined enhancement of the tumor and FGT, (f) texture of FGT enhancement, (g) texture of tumor enhancement, (f) spatial heterogeneity related to tumor enhancement, (h) variations in FGT enhancement, and (i) variations in tumor enhancement” [24].

### Workflow

In this study, we adopted three different models:

1. Clinical model: Focused on utilizing machine learning algorithms with exclusively clinical and pathology data. This model leveraged various patient-specific factors, such as demographics, medical history, and histopathological characteristics, to develop predictive models for recurrence.
2. Radiomic model: Centered on machine learning algorithms specifically designed to examine aspects of dynamic contrast-enhanced magnetic resonance imaging (DCE-MRI) features. By using a comprehensive set of quantitative imaging features, we aimed to capture subtle characteristics of the tumor and its surrounding tissues that may provide insights into the likelihood of recurrence.
3. Merged model: Aimed to combine the strengths of the clinical and radiomic data by integrating both datasets. This model allowed us to harness the complementary information provided by the clinical factors and the detailed imaging features, creating a more comprehensive predictive model for breast cancer recurrence.

The software environment that we utilized in our research, which consists of Python 3.10.12, scikit-learn 1.2.2, XGBoost 2.0.3, and imbalanced-learn 0.10.1, provides cancer centers with a useful option to utilize machine learning’s predictive capabilities when it comes to breast cancer recurrence. As Python is an accessible and flexible programming language, cancer centers may easily incorporate these models into their current computational settings. The scikit-learn framework is a solid base upon which a variety of machine learning methods may be implemented, giving cancer centers an intuitive interface through which to modify and expand our models. XGBoost is a powerful and effective toolkit that excels in managing complicated datasets, which is especially useful for oncology-related applications. Our dedication to tackling the difficulties presented by imbalanced datasets, which are frequently encountered in clinical settings, is demonstrated by the addition of imbalanced-learn, which guarantees that the models retain accuracy even when exposed to unequal class distributions. Cancer centers can begin by gathering pertinent clinical and pathological data from patient records in order to apply these models. A strong basis is provided by utilizing the scikit-learn library for Logistic Regression, Adaptive Boosting, and Random Under-sampling Boosting, as well as XGBoost for Extreme Gradient Boosting. Specifically, imbalanced datasets that are frequently encountered in clinical contexts are addressed by the implementation of RUSBoost using imbalanced-learn. Preprocessing steps involve cleaning and normalizing the collected data, and integration of dynamic contrast-enhanced magnetic resonance imaging (DCE-MRI) characteristics facilitates a comprehensive approach.

Clinicians can interpret model outputs, including receiver operating characteristic curves, precision, recall, and F1 score, to inform decision-making. Regular retraining using updated datasets is recommended to ensure continued accuracy, and collaboration with the IT department aid seamless integration into existing clinical workflows. By building upon our results, cancer centers have the opportunity to integrate advanced machine learning techniques seamlessly into their clinical workflows, ultimately contributing to more accurate predictions and improved patient care.

**Fig. 2:**
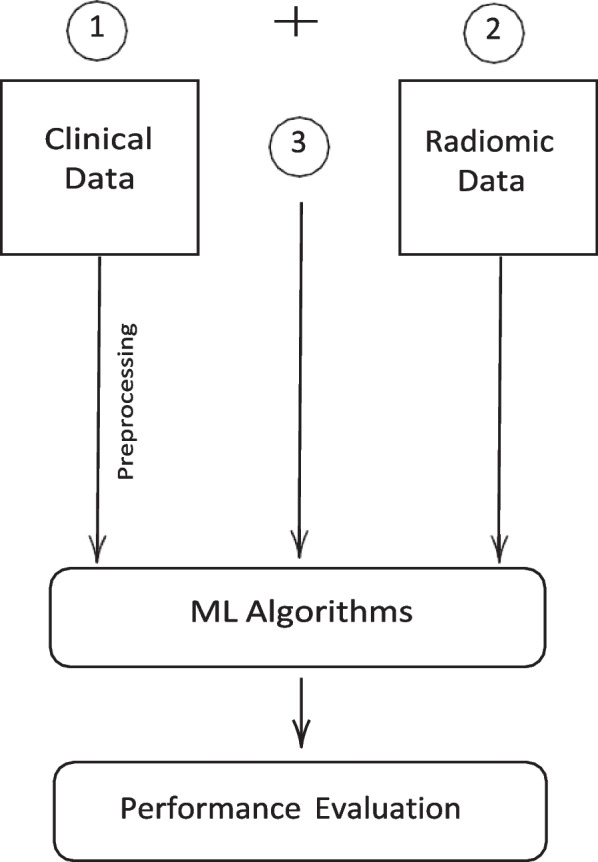
Workflow of the study

A flowchart (Fig. 2) was utilized to illustrate the methodology employed in our research. The first path (1) represents the clinical model, wherein we preprocessed the clinical data and subsequently applied machine learning (ML) algorithms, which will be explained in detail later. In the second model (2) and the third model (3), ML algorithms were applied to the radiomic data and merged data, respectively. Finally, we evaluated the performance of all three models using various evaluation metrics, which will be discussed in greater detail. In this study, five machine learning techniques were put into practice: Adaptive Boosting (AdaBoost), Random Under-sampling Boosting (RUSBoost), Extreme Gradient Boosting (XGBoost), support vector machines (SVM), and Logistic Regression. These algorithms were chosen for their effectiveness in addressing classification and regression problems.

- *SVM*: The support vector machines family of machine learning allows for the resolution of classification, regression, and anomaly detection problems. They are renowned for their strong theoretical guarantees, high flexibility, and ease of use even for those with no prior knowledge of data mining [26].
- *Logistic Regression*: Is a statistical model that enables the study of the relationship between a group of qualitative variables Xi and a qualitative variable Y. It also allows for predicting the likelihood of an event based on the regression coefficient [27].
- *XGBoost*: Is an ensemble learning method that employs a gradient reinforcement framework, is built on an ensemble decision tree, and is based on the sequential self-improvement principle; it works sequentially to improve by capitalization in comparison to previous execution [28].
- *RUSBoost*: Is a variant of SMOTEBoost, designed specifically to address the issue of data class imbalance. It employs a combination of data sampling and boosting to reduce majority class sampling and to more accurately predict the minority class [29].
- *AdaBoost*: Similar to XGBoost, it generates sequentially weak learners. This technique attempts to improve the system by focusing on misclassified data [30].

### Evaluation metrics

Imbalanced datasets pose a significant challenge for machine learning models due to the unequal distribution of classes, leading to difficulties in accurately predicting the minority class. This issue is commonly encountered in various health applications [31], including our study. Specifically, our dataset exhibited a higher number of instances representing the non-recurrence event class compared to the recurrence event class. Consequently, evaluating the performance of machine learning algorithms on this dataset requires the utilization of evaluation metrics that effectively address the problem of imbalanced data distribution.

The metrics used in this study are as follows:

#### Precision and recall

Are essential metrics in classification because of their robustness and interpretability. They are based on the confusion matrix and focus on the performance of the model on positive individuals:

*Precision:* Is also known as positive predictive value, and it measures the model’s ability to avoid making an error during a positive prediction. It corresponds to the success rate of positive predictions.

$$Precision= \frac{True\;positive}{True\;positive\;+\;false\;positive}$$

*Recall:* Is sometimes referred to as sensitivity, true-positive rate, or hit rate. It measures the model’s ability to identify all positive individuals and is correlated with the model’s detection rate of positive individuals.

$$\mathrm{Recall}=\frac{\mathrm{True}\;\mathrm{positive}}{\mathrm{True}\;\mathrm{positive}\;+\;\mathrm{false}\;\mathrm{negative}}$$

#### F1 score

It is the average of recall and precision, which shows how recall and precision can be combined to produce a higher rating. Consequently, it performs well on an imbalanced dataset.

$${\mathrm{F}}1\;\mathrm{ score}=\frac{2}{{}^{1}\!\left/ \!{}_{{\mathrm{Precision}}}\right.+{}^{1}\!\left/ \!{}_{{\mathrm{Recall}}}\right.}$$

#### ROC curve

ROC is the abbreviation of “receiver operator characteristics”. It is used to evaluate the effectiveness of various model classifications from one or more models. The higher the value of a curve, the larger the area under the curve, the less error the classifier makes.

#### AUC

It is an acronym for “area under the ROC curve.” It is a synopsis of the ROC curve that shows the degree to which a classifier is capable of differentiating across classes. The model performs better at differentiating between positive and negative classes the higher its AUC.

## Results

The results obtained from the first, second, and third model are illustrated in the tables below (Tables 1, 2, 3).

**Table 1:** Classification report for clinical data

	Precision	Recall	F1 score	AUC
SVM	0.738	0.742	0.740	0.814
Logistic regression	0.841	0.840	0.841	0.898
XGBoost	0.946	0.887	0.915	0.954
RUSBoost	0.912	0.887	0.899	0.914
AdaBoost	0.915	0.885	0.900	0.915

**Table 2:** Classification report for radiomic data

	Precision	Recall	F1 score	AUC
SVM	0.822	0.853	0.837	0.912
Logistic Regression	0.775	0.869	0.819	0.877
XGBoost	0.960	0.968	0.964	0.994
RUSBoost	0.864	0.916	0.890	0.943
AdaBoost	0.864	0.916	0.890	0.943

**Table 3:** Classification report for merged data

	Precision	Recall	F1 score	AUC
SVM	0.958	0.928	0.942	0.994
Logistic Regression	0.996	0.991	0.993	0.995
XGBoost	0.988	1	0.993	0.995
RUSBoost	0.769	0.747	0.758	0.800
AdaBoost	0.884	0.887	0.885	0.959

**Fig. 3:**
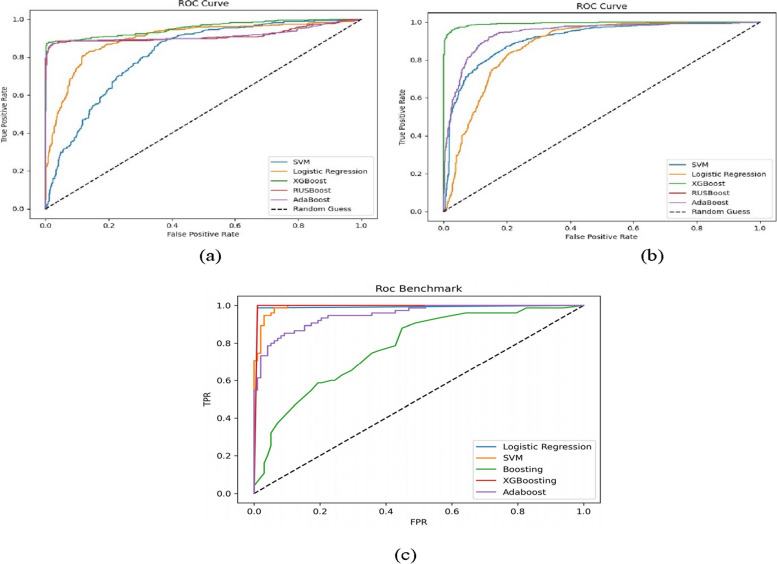
ROC benchmarks for different algorithms using the three models. **a** Clinical model. **b** Radiomic model. **c** Merged model

Figure 3 presents the ROC curve benchmark for the different algorithms using the three models.

### Using clinical model

As shown in Table 1, when considering the clinical data alone, the results show that Logistic Regression performed consistently well, achieving high precision, recall, F1 score, and AUC. SVM also demonstrated competitive performance across these metrics. XGBoost, on the other hand, outperformed other algorithms in terms of precision and achieved a high AUC score, indicating its effectiveness in leveraging clinical data for prediction.

The AUC values obtained are translated in Fig. 3a. The SVM algorithm achieved an AUC of 0.814, indicating a reasonably good performance in distinguishing between the two classes (recurrence and non-recurrence) based on clinical data. The Logistic Regression algorithm performed better with an AUC of 0.898, suggesting a higher discriminatory ability. XGBoost and the ensemble methods (RUSBoost and AdaBoost) demonstrated even stronger performance, achieving AUC values of 0.954 and 0.914–0.915, respectively.

### Using radiomic model

For the radiomic data (Table 2), XGBoost emerged as the top-performing algorithm, demonstrating the highest precision, recall, F1 score, and AUC among all the evaluated algorithms. This suggests that radiomic characteristics taken out of medical scans hold significant predictive power for breast cancer recurrence. However, it is worth noting that SVM and Logistic Regression also achieved notable performance in terms of precision, recall, and AUC on the radiomic data.

When analyzing the ROC curves for the radiomic model, we can gain valuable insights into the classification performance and discriminatory ability of the algorithms in predicting breast cancer recurrence (Fig. 3b). The SVM algorithm showed improved performance on radiomic data, achieving an AUC of 0.912. Logistic regression also demonstrated relatively good discrimination with an AUC of 0.877. Notably, XGBoost performed exceptionally well on radiomic data, yielding an AUC of 0.994. This high AUC value indicates a robust ability to classify instances using radiomic features. RUSBoost and AdaBoost achieved AUC values of 0.943, indicating good performance but slightly lower than XGBoost.

### Using the merged model

Interestingly, when the clinical and radiomic data were merged, the performance of the algorithms improved further (Table 3). SVM and XGBoost achieved exceptionally high precision, recall, F1 score, and AUC, indicating the complementary nature of the two types of data. Logistic regression achieved near-perfect precision and recall, demonstrating the potential of merged data for accurate prediction of breast cancer recurrence. While RUSBoost and AdaBoost showed competitive performance in some cases, their results were generally overshadowed by the other algorithms in terms of precision, recall, and F1 score. It is important to consider these results in the context of the specific dataset and the choice of evaluation metrics. Upon analyzing the ROC curves for the merged model (Fig. 3c), it is apparent that the combination of clinical and radiomic data yields significantly higher AUC values compared to the utilization of each data type independently. SVM achieved an impressive AUC of 0.994, suggesting excellent discrimination capabilities when using merged data. Logistic regression achieved an AUC of 0.995, indicating near-perfect separation of the two classes. XGBoost maintained its outstanding performance with an AUC of 0.995 on the merged data. RUSBoost and AdaBoost achieved lower AUC values of 0.800 and 0.959, respectively, indicating moderate to good discriminatory ability on the merged data.

## Discussion

The results of this study illustrate the capacity to improve the predictive accuracy for breast cancer recurrence by integrating clinical and radiomic data. The study employed three distinct models, specifically the clinical model, radiomic model, and merged model, to yield significant findings regarding the efficacy of employing diverse data types.

The clinical model, which exclusively relied on clinical and pathology data, demonstrated a moderate level of predictive performance across all evaluation metrics. The performance of the SVM and Logistic Regression algorithms exhibited similar results in terms of precision, recall, F1 score, and AUC. Nevertheless, their performance was lacking in comparison to the other algorithms.

On the other hand, the radiomic model, which solely employs DCE-MRI imaging features, exhibited a notable enhancement in its predictive capability. The XGBoost algorithm demonstrated superior performance compared to the other algorithms, exhibiting high levels of precision, recall, and F1 score. Furthermore, the model demonstrated a noteworthy AUC, suggesting its proficiency in accurately distinguishing between cases of recurrence and non-recurrence. This underscores the significance of integrating radiomic features into the prediction of breast cancer recurrence.

The model that was created by merging clinical and radiomic data demonstrated superior predictive performance in contrast with the other two models. The support vector machine (SVM) algorithm demonstrated high levels of precision, recall, and F1 score, whereas the Logistic Regression and XGBoost algorithms also exhibited strong performance. The area under the receiver operating characteristic curve (AUC) values for all algorithms within the merged model exhibited a significant increase compared to the AUC values observed in the other models. This notable improvement in AUC values suggests an enhanced ability to discriminate between different classes within the merged model.

Examining the ramifications of our ROC benchmark results (Fig. 3) in the context of actual clinical practice allows us to gain important knowledge that will direct future investigations and improve patient care. The combined model’s discriminative ability, which incorporates radiomic and clinical data, shows great promise for converting machine learning breakthroughs into useful therapeutic uses. The excellent rise in area under the curve (AUC) values highlights the combined model’s strong capacity to differentiate between breast cancer recurrence and non-recurrence. This is especially true when considering that Logistic Regression and XGBoost achieve nearly complete separation at 0.995. This increased precision results in a more accurate identification of patients who are at risk, which is a crucial component of clinical decision-making. In real-world clinical scenarios, the support vector machine (SVM), Logistic Regression, and XGBoost algorithms within the merged model demonstrate exceptional precision, recall, and F1 scores. These metrics underscore the reliability of the model in accurately identifying cases of recurrence while minimizing false positives and negatives. Such precision is paramount in clinical decision-making, facilitating timely interventions and personalized treatment strategies. Furthermore, the impressive performance of the merged model aligns closely with the nuanced and dynamic status of breast cancer patients, making it a valuable asset for clinicians. The ability to integrate diverse data types, including clinical information and radiomic features, strengthens the model’s applicability in diverse patient populations with varying disease kinds and stages. This adaptability is crucial in addressing the inherent heterogeneity within clinical cohorts, enhancing the model’s generalizability and relevance in routine clinical practice. As we navigate the complex landscape of breast cancer management, our findings advocate for the seamless integration of machine learning models, particularly the merged model, into the clinical workflow. This approach has the potential to augment existing diagnostic and prognostic tools, aiding oncologists in making more informed decisions and improving patient outcomes. We encourage researchers to build upon these findings, refining and expanding the models to further align with the intricacies of real-world patient scenarios. By fostering collaborations between data scientists and clinical practitioners, we can bridge the gap between cutting-edge research and tangible improvements in breast cancer care.

The aforementioned findings indicate that the amalgamation of clinical and radiomic data has the potential to offer a more profound comprehension of the prediction of breast cancer recurrence. The integration of individualized clinical data with quantitative imaging characteristics improves the efficacy and reliability of predictive models.

The selection of machine learning algorithms had a substantial impact on the predictive capabilities of all three models. It is worth mentioning that XGBoost consistently demonstrated superior performance as an algorithm, showcasing its resilience in effectively managing both radiomic and clinical data. The versatility and effectiveness of XGBoost in capturing complex patterns and relationships within the dataset are highlighted by its exceptional performance across all models [32].

The incorporation of varied research, encompassing studies beyond the realm of breast cancer, amplifies the significance of our discoveries and provides invaluable perspectives into the wider field of predictive modeling in oncology. Specifically, research on endometrial cancer [33], rectal cancer [34], and hepatocellular carcinoma [35] highlights the common theme among these malignancies: the ability to combine radiological and clinical data for better predictive accuracy.

An intriguing study [36] highlights the importance of integrating dual-view radiomics with clinical characteristics in the context of breast cancer, demonstrating superior performance in malignancy evaluation. This is consistent with our methodology, in which the combination of radiomic and clinical variables produced increased discriminatory power, highlighting the need of a thorough approach in the prediction modeling of breast cancer.

Additionally, current research explores the use of radiomic and clinical features in predicting recurrence for patients with breast cancer. The trend of merging various data types for predictive modeling is mirrored in the combination of machine learning analyses and [18F]-FDG-PET-based radiomic characteristics [22]. The idea that radiomic signatures improve prognosis prediction accuracy is supported by a different study that focuses on triple-negative breast cancer (TNBC) and demonstrates the complementing role of radiomics in predicting recurrence-free survival [37].

By contrasting our findings with those of similar studies, we provide strong evidence for the efficacy and generalizability of the combined clinical and radiomic method in predictive modeling of various cancer types. The combined results highlight the potential translational significance of our research and highlight how a multimodal approach can always be useful in guiding clinical decision-making and enhancing patient outcomes.

It is important to acknowledge that our study employed a diverse cohort of patients, who underwent scanning in various ways, representing different stages and types of cancer, including both triple-negative and non-triple-negative breast cancer. This contributes to the overall transfer ability and practicality of the results to a wide range of patient cohorts.

Additional research is necessary to substantiate the findings using larger and more diverse datasets, as well as to investigate supplementary data sources and advanced machine learning methodologies.

In general, the findings of this study offer significant contributions to the field of research and clinical practice, particularly in the context of enhancing the forecast for the return of breast cancer. Radiation therapy professionals have a great chance to customize treatment and make informed choices through the use of machine learning approaches to construct prediction models. In the context of managing breast cancer recurrence, medical personnel can improve patient outcomes by using integrated clinical and radiomic data to inform their decisions and potentially improve patient well-being overall.

## Conclusion

In summary, this research adds to the expanding collection of scholarly works on the prediction of breast cancer recurrence and highlights the promise of incorporating clinical and radiomic data to further the field of personalized medicine. Through the utilization of machine learning algorithms, there exists the potential to advance towards enhanced precision and personalized methodologies in the prevention and control of breast cancer recurrence.

The implications of the study’s findings hold significant importance for subsequent research endeavors and clinical applications within the realm of breast cancer recurrence prediction. The amalgamation of clinical and radiomic data, in conjunction with the judicious choice of machine learning algorithms, has the potential to enhance the accuracy of treatment planning and decision-making in the field of radiation therapy. Predictive models present valuable resources for the field of personalized medicine, holding the potential to enhance patient outcomes through the identification of individuals with an elevated risk of recurrence. Overall, the ongoing progress of artificial intelligence and its incorporation into the field of medical physics exhibits potential for enhancing decision-making in radiation therapy and optimizing the overall management of breast cancer recurrence.

## Data Availability

The datasets generated and/or analyzed during the current study are available in the The Cancer Imaging Archive [24] repository, https://wiki.cancerimagingarchive.net/pages/viewpage.action? pageId = 70,226,903#702269035253f6ef81dd49c486558e45d1dc5d75 [23].

## Abbreviations

AdaBoost: Adaptive Boosting
AUC: Area under the receiver operating characteristic curve
DCE-MRI: Dynamic contrast-enhanced magnetic resonance imaging
ER: Estrogen receptor
FDG-PET: Fluorodeoxyglucose-positron emission tomography
FGT: Fibroglandular tissue
HER2: Human epidermal growth factor receptor 2
KNN: K-nearest neighbor
ML: Machine learning
nCV: Nested cross validation
Non-TNBC: Non-triple-negative breast cancer
PLS: Partial least square
PR: Progesterone receptor
ROC: Receiver operating characteristic curve
RUSBoost: Random Under-sampling Boosting
SMOTE: Synthetic minority oversampling technique
SVM: Support vector machine
TCIA: The Cancer Imaging Archive
TNBC: Triple-negative breast cancer
XGBoost: Extreme Gradient Boosting
